# Self-administered physical exercise training as treatment of neck and shoulder pain among military helicopter pilots and crew: a randomized controlled trial

**DOI:** 10.1186/s12891-017-1507-3

**Published:** 2017-04-07

**Authors:** Mike Murray, Britt Lange, Bo Riebeling Nørnberg, Karen Søgaard, Gisela Sjøgaard

**Affiliations:** 1 Department of Sports Science and Clinical Biomechanics, University of Southern Denmark, Odense, Denmark; 2 Department of Anesthesia and Intensive Care Medicine, Odense University Hospital, Odense, Denmark; 3 Royal Danish Air Force, Air Force Staff, Defence Command Denmark, Karup, Denmark

**Keywords:** Musculoskeletal disorders, Neck, Pain, Exercise, Pilot, Crew-member, Clinical trial, Intervention, Pressure-pain-threshold

## Abstract

**Background:**

Neck pain is frequent among military helicopter pilots and crew-members, and pain may influence individual health and work performance. The aim of this study was to examine if an exercise intervention could reduce neck pain among helicopter pilots and crew-members.

**Methods:**

Thirty-one pilots and thirty-eight crew-members were randomized to either an exercise-training-group (*n* = 35) or a reference-group (*n* = 34). The exercise-training-group received 20-weeks of specific neck/shoulder training. The reference-group received no training. Primary outcome: Intensity of neck pain previous 3-months (scale 0-10). Secondary outcomes: additional neck/shoulder pain intensity variables and pressure-pain-threshold in the trapezius muscle (TRA) and upper-neck-extensor muscles (UNE). Regular training adherence was defined as ≥1 training session a week. Statistical analyses performed were intention-to-treat and per-protocol. Students *t*-test was performed (*p* < 0.05).

**Results:**

Intensity of neck pain previous 3-months at baseline was: 2.2 ± 1.8 and previous 7-days: 1.0 ± 1.5, and pressure-pain-threshold in TRA and UNE (right/left) was in kPa: 424 ± 187 / 434 ± 188 and 345 ± 157 / 371 ± 170 in the exercise-training-group, and 416 ± 177 / 405 ± 163 and 334 ± 147 / 335 ± 163 in the reference-group, with no differences between groups. Intention-to-treat-analysis revealed no significant between-group-differences in neck pain intensity and pressure-pain-threshold. Between-group-differences, including participants who trained regularly (*n* = 10) were also non-significant. Within-group-changes were significant among participants with regular training adherence in the exercise-training-group regarding intensity of neck pain previous 3-months (from 2.2 ± 0.6 to 1.3 ± 1.3, *p* = 0.019). Likewise, within the whole exercise-training-group, neck pain previous 7-days decreased (from 1.0 ± 1.4 to 0.6 ± 1.1, *p* = 0.024). Additional within-group-changes regarding pressure-pain-threshold in kPa were for the reference-group a reduction in TRA and UNE (right/left) to: 342 ± 143 / 332 ± 154 and 295 ± 116 / 292 ± 121 implying increased pain sensitivity, while for the exercise-training-group only a reduction in left TRA was seen: 311 ± 113.

**Conclusions:**

The exercise intervention did not reduce neck pain among helicopter pilots and crew-members as no significant between-group-differences were found. However, some trends were demonstrated as some neck pain intensity and sensitivity improved more within the exercise-training-group but not within the reference-group. The lack of effect may be due to low adherence since only ~ 1/3 of subjects in the exercise-training-group engaged in regular training which may be due to the self-administration of the training.

**Trial registrations:**

Ethical committee of Southern Denmark (S-20120121) 29 August, 2012. Clinical Trail Registration (NCT01926262) 16 August, 2013.

## Background

Neck pain among helicopter pilots and crew-members is a challenging problem within modern air forces [1]. The 3-months prevalence of neck pain among helicopter pilots has previously been estimated to 57% with 32% of pilots reporting recurrent pain episodes [2]. These prevalence rates are high compared to a 6-months prevalence of ~ 30% and a 1-year prevalence of ~ 40% within the general working population [3]. Neck pain represents an individual health concern at leisure and may as well influence pilots and crew-members level of concentration [4], thereby potentially affecting operational safety. Neck pain may also impact operational capacity within air forces through increased sickness leave [1]. Limited research has been conducted on neck pain among helicopter pilots and especially crew-members. With some exceptions, due to work tasks [5], helicopter pilots and crew-members undergo similar in-flight exposures such as the use of a flight helmet and helmet mounted devices like night vision goggles (NVG). Especially loading of the cervical spine has been reported as a risk factor for neck pain and discomfort in pilots and crew-members [6]. Loading of the cervical spine has also been measured in laboratory settings combining different head positions [7, 8], and during real flight [9]. Based on current results loading of the cervical spine poses a heavy burden on the cervical spine musculature of pilots as well as crew-members which may potentially result in neck pain episodes. It is therefore imperative that both groups are addressed regarding flight related neck pain. Physical exercise training has been found effective as a deterrent against neck pain in a number of large intervention studies conducted within other working populations [10–12]. Specifically tailored exercise interventions have proven particularly effective [13, 14]. Few exercise interventions have been conducted among helicopter pilots and crew-members with successful outcome regarding reduction of neck pain. Theoretically, exercise training may increase individual capacity and reduce the relative workload on the cervical musculature, thereby reducing the risk of developing neck pain [15]. However, no currently evidence-based guidelines, contributing to the prevention and clinical management of neck pain among helicopter pilots and crew, exist within the Royal Danish Air Force (RDAF). It is therefore important that further exercise studies are conducted in order to increase available knowledge.

The aims of this study were to 1) determine the prevalence of neck and shoulder pain among military helicopter pilots and crew-members within the RDAF, and to 2) evaluate the effectiveness of a 20-week exercise intervention on intensity of neck pain among helicopter pilots and crew-members. The hypothesis tested was that the exercise intervention resulted in a reduction in self-reported intensity of neck pain previous 3-months. Secondarily, it was tested if objectively measured pain sensitivity in the neck and shoulder muscles as well as other neck/shoulder pain intensity variables were reduced.

## Methods

### Design of the study

The study design was a parallel group, single blinded, randomized, controlled trial. The intervention period was 20-weeks with pre-intervention baseline measurements and post-intervention follow-up measurements. A detailed protocol paper has been published previously [13]. The trial was conducted within the RDAF from November 2013 to April 2014. All participants volunteered and gave their written informed consent before participation. The trial was approved by the local Ethics Committee of Southern Denmark (S-20120121) and qualified for registration in ClinicalTrials.gov (NCT01926262).

### Participants and randomization

**Fig. 1:**
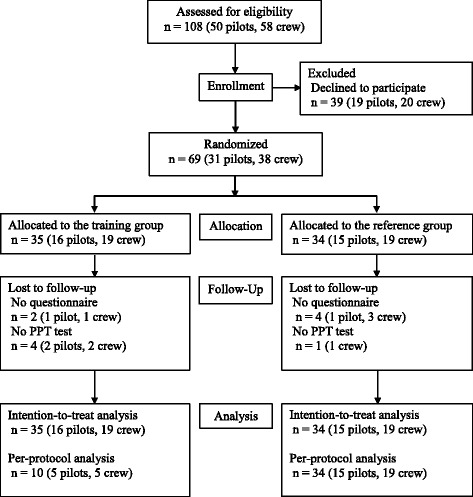
Flow diagram

Fifty military helicopter pilots and fifty-eight crew-members from two different RDAF squadrons were invited to participate in the study. Participants were informed about the project at briefings, by email, and by telephone. Thirty one pilots (2 female and 29 male) and thirty-eight crew-members (male) agreed to participate. Inclusion criteria comprised: 1) profession as a helicopter pilot or crew-member (technician, systems operator, tactical helicopter observer and/or navigator), 2) maintaining operational flight status at enrollment, 3) operational flying within the previous 6-months. Exclusion criteria comprised: 1) participation in a training intervention during the previous 12-months. Participants flow is depicted in Fig. 1. Participants were assigned a random identification number at enrollment by an authorized person with no relation to the study. After pre-intervention assessments participants were randomized 1:1 to either an exercise-training-group (ETG) or to a reference-group (REF). Participants were stratified according to the following nested criteria to ensure comparability between the ETG and REF: 1) squadron (722 squadron or 724 squadron), 2) profession (pilot or crew-member), 3) age (< or ≥ 40 years of age), and 4) flying experience (< or ≥ 2500 h). The random identification numbers within each stratum were drawn from an opaque, tossed bag. Alternately, the first number in the first strata was allocated to either the ETG or REF depending on the flip of a coin. The first number in the second strata was allocated to the opposite group, compared to the last number in the previous strata, and so forth. The randomization procedure was carried out by a blinded custodian (last author) using the random identification numbers assigned to the participants. Data analysts and statistician were blinded to the random group allocation of the participants.

### Measurements

All methods have been previously described [13], and will therefore only be explained briefly. Anthropometric measurements included: height, seated height, weight and body mass index (BMI) (Composition Analyzer Tanita Corporation of America, USA).

Participants’ pressure-pain-threshold (PPT) was measured in the neck and shoulder muscles. PPT was measured bilaterally for the trapezius muscle (TRA) (20% medially to half the distance between the lateral edge of the acromion and seventh cervical vertebra) [16], the upper neck muscles (UNE) (2 cm laterally to the vertical line of the axis in level with the 4’th cervical vertebra) [17, 18], and the anterior tibialis muscle (TIA) (as the point of reference) [19]. A handheld electronic pressure algometer was used (Type II Algometer, Somedic Production AB, Sweden). The algometer was pistol-shaped with a pressure-sensitive strain gauge at the tip. The contact area had a diameter of 1 cm^2^. Compression pressure was applied perpendicularly to the skin with a rate of 20 kPa/s. A digital display on the pressure algometer was used to keep the rate of pressure stable. Measurements were performed three times in a fixed order: 1) right TRA, 2) left TRA, 3) right UNE, 4) left UNE, and 5) right TIA. A rest period of approximately 1 min was given between measurements conducted on the same PPT point. Participants were given a hand held control switch and were instructed to immediately press the switch when the sensation of “pressure” changed to “pain”. When the switch was pushed the compression was stopped and the pressure was released [20]. A low level of pain sensitivity therefore equals a high PPT value, and a high level of pain sensitivity equals a low PPT value. The maximum applied pressure registered was recorded before resetting the algometer. A maximal pressure of 1000 kPa was allowed for TRA and TIA and 700 kPa for the UNE. The algometer was calibrated before each test. Measurements were conducted by an experienced sports scientist.

### Questionnaire

An online based questionnaire was applied to participants pre- and post-intervention. The questionnaire was confidential using the assigned identification numbers. A modified version of the validated Nordic Musculoskeletal Questionnaire [21] was used to assess the prevalence and intensity of musculoskeletal symptoms in the following body regions: neck and shoulders, upper back, elbows, low back, wrists/hands, hips/thighs, knees, and ankles/feet according to: 1) the number of days with pain or complaints in (body region) the previous 12-months (possible answers: 0 days, 1 – 7 days, 8 – 30 days, 31 - 90 days, > 90 days, or every day), 2) inability to perform daily working tasks due to complaints in (body region) the previous 3-months (possible answers: yes or no), 3) intensity of (body region) pain previous 3-months was assessed on a scale from 1-10 (10 = worst possible pain imaginable) and to this was added 0 = no pain, resulting overall in an 11 point numeric box scale, and 4) intensity of (body region) pain previous 7-days depicted on the same box scale. All questions were accompanied by chart illustrations of the body region in focus. Furthermore, participants were inquired about the amount of total flying hours in fixed-wing aircraft, rotary-wing aircraft, and flying hours with NVG. Lastly, the questionnaire included a number of health and work related questions [13].

Participants randomized to the ETG received an additional questionnaire regarding: 1) their motivation to train, 2) expectations, 3) training adherence, and 4) adverse training effects. Training adherence was measured by inquiring: “You were instructed to train 3x20 min a week. How did you succeed?” Possible answers: “I trained regularly 2-3 times a week, I trained regularly 1-2 times a week, I trained irregularly, but at least 4 times a month (approximately once a week), I trained irregularly but at least 2-3 times a month, I trained some, but stopped training after a while, and I did not use the training offer”.

### Exercise intervention

Participants in the ETG received 20-weeks of strength, endurance, and coordination training targeting the neck and shoulder muscles. Training was divided into training sessions of 3x20 min per week. The training programme was evidence based and designed by an interdisciplinary team of sports exercise training specialists, physiotherapists, doctors and chiropractors. The training programme was composed of ten training exercises divided into three categories: 1) two warming up exercises, 2) six neck exercises, and 3) two shoulder exercises. All exercises have been described previously [13] and training videos are available online [22].

Every training session was initiated with one or two warming up exercises recruiting the deep cervical muscle groups. Exercises included: cervical flexion from a supine position and cervical rotation from an erect position. The warming up exercises were performed using 3 sets of 15 repetitions. Intensity was increased as participants progressed. These two exercises also aimed to warm up the neck before engaging in more strenuous training exercises. The warming up exercises were followed by training exercises for the larger neck muscles including: cervical extension, cervical flexion (straight forward and oblique angels), and lateral flexion. Finally, participants performed two exercises for the shoulder girdle including: shrugs and reverse flies. Neck and shoulder exercises were performed using elastic training bands for resistance (Thera-Band®, The Hygenic Corporation, USA). The training program was designed to be progressive using an undulating design with sets ranging between 2-4 and training intensity ranging between 12 – 20 repetitions depending on week of training. The training equipment used was light weight to allow for easy transportation when participants traveled between Air Force Bases. Participants received a training bag including: a head harness (Neck Flex, Gonzo Companies, USA), six color-coded levels of resistance bands (red, green, blue, black, silver and gold), exercise handles, and a door anchor (Thera-Band®, The Hygenic Corporation, USA), and a training manual that described all training exercises in detail. In addition, participants were given online access to a training homepage with supplementary training information and training videos for each exercise. All participants received a personal training diary that described when to perform the various training exercises. Training was to be performed within working hours or if preferred at leisure.

### Training adherence

Training was based on self-management education due to a dynamic work schedule among participants and frequent travel between Air Force Bases. At the beginning of the intervention all participants received an individual or group introduction to the training program. The introduction included: 1) a detailed description of the training program and diary, 2) an introduction to all training exercises and adjustments to ensure high quality on exercise performance, and 3) practical information regarding supervision during the intervention period. Participants received at least one follow-up visit during the intervention period in order to make sure that training exercises were performed correctly, and with progression. To motivate participants in the ETG to train, motivational posters were hung on the walls in the rooms of the two squadrons and tweets were posted on the training homepage. Thus, the training was not regularly supervised but was self-administered with the above support actions.

### Statistical analysis

A pre-intervention power analysis was performed on the single primary outcome variable of self-reported intensity of neck pain previous 3-months [13]. Pain intensity was rated on an 11 point numeric box scale. The analysis showed that we would need to include 54 participants (27 experimental subjects and 27 control subjects) in this study. The analysis was based on the finding that a change of 1 measured on a 11 point numeric box scale is considered the minimum clinically significant difference regarding change in pain [23]. We also used results on pain intensity from a previous study among military pilots that found the response within this subject group to be normally distributed with a standard deviation in neck pain intensity of 1.5 the previous 3-months [24]. With a power set at 0.8 and a probability of a type I error of *p* < 0.05, we would be able to detect a true difference in mean response of neck pain between experimental and control subjects equal to ± 1.2 measured on an 11 point numeric box scale. Allowing for a 10% loss to follow-up, the total number of participants required was 64. The null-hypothesis (no difference between experimental and control subjects) was to be rejected if a between-group-difference for intensity of neck pain previous 3-months was significant (*p* < 0.05). The relationship between intensity of neck and shoulder pain previous 3-months, and pilots’ and crew-members: age, height, sitting height, weight, BMI, flying hours in fixed-wing aircraft, flying hours in rotary-wing aircraft, and flying hours with NVG, was analysed by multiple regression. Two statistical analyses were performed: 1) an intention-to-treat analysis comparing participants in ETG and REF as originally allocated after randomization [25], and 2) a per-protocol analysis only including participants in ETG that adhered regularly to the exercise intervention. Regular training adherence was defined as training between 1–3 times a week during the 20-week intervention period (≥ 33.3% of the total amount of training sessions). For missing values last observation carried forward or backwards were imputed. If observations were missing at both baseline and follow-up, the population mean was imputed at baseline, and group mean (ETG or REF) at follow-up. Between-group-differences at baseline were analyzed using the Student’s *t*-test. The same analysis was performed at follow-up, including an analysis on delta-values (calculated by subtracting the pre- from post-intervention values). Within-group-changes were analyzed by a paired *t*-test. The level of statistical significance was *p* < 0.05. Results are presented as sample means and standard deviations (mean ± SD) if not otherwise specified. Statistical analyses were performed in Stata Statistics/Data Analysis version 13.0 (StataCorp LP, USA).

## Results

### Pre-intervention

#### Participant characteristics

Overall mean (±SD) for all participants was for age: 40 years (40.6 ± 7.6 years), height: 1.81 m (1.81 ± 0.1 m), and weight: 84 kg (83.9 ± 12.2 kg). No significant pre-intervention differences pertaining to anthropometric values or flight experience were identified between the ETG and REF (Table 1).

**Table 1:** Participants’ characteristics

	ETG (*n* = 35)	REF (*n* = 34)	*p*-value
Age (years)	40.4 ± 6.7	40.7 ± 8.4	0.880
Height (m)	1.82 ± 0.07	1.80 ± 0.08	0.360
Seated height (m)	0.95 ± 0.05	0.95 ± 0.04	0.995
Weight (kg)	84.2 ± 12.7	83.7 ± 11.8	0.882
Body mass index (BMI)	25.4 ± 3.0	25.7 ± 2.3	0.640
Flying hours in fixed-wing aircraft (hours)	226.3 ± 565.3	124.1 ± 371.5	0.523
Flying hours in rotary-wing aircraft (hours)	1778.1 ± 1214.3	2142.8 ± 1451.1	0.374
Flying hours with NVG (hours)	160.6 ± 99.1	174.6 ± 142.0	0.995

Values are presented as mean ± SD. Exercise-training-group (ETG), Reference-group (REF), Night vision goggles (NVG)

#### Pain prevalence

The 12-month prevalence of neck pain was 90.3% for helicopter pilots. Of these 54.8% had experienced 1-7 days with neck pain, 32.3% had experienced 8-30 pain days, and 3.2% had experienced > 90 days with neck pain. For crew-members the 12-month prevalence of neck pain was 81.6%. Of these, 44.7% had experienced 1-7 pain days, 29.0% had experienced 8-30 pain days, 2.6% had experienced > 90 pain days, and 5.3% had experienced neck pain daily. The 12-month prevalence of shoulder pain (right/left side) was 54.8% / 32.3% for pilots, and 42.1% / 39.5% for crew. No significant pre-intervention differences were found between the ETG and REF group regarding pain intensity, neither in the neck nor the shoulder within the previous 3-months or 7-days (Table 2).

**Table 2:** Intention-to-treat analysis of neck and shoulder pain intensity the previous 3-months and 7-days

	ETG (*n* = 35)	REF (*n* = 34)	(95% Conf. Interval)	*p*-value
Neck pain previous 3-months				
Pre-intervention	1.9 ± 1.7	2.5 ± 1.9	- 0.6 (- 1.5 - 0.3)	0.159
Post-intervention	1.9 ± 2.0	1.9 ± 1.8	- 0.1 (- 1.0 – 0.8)	0.865
Change	0.0 ± 2.5	- 0.5 ± 1.7	0.5 (- 0.5 – 1.6)	0.291
Neck pain previous 7-days				
Pre-intervention	1.0 ± 1.4	1.0 ± 1.5	0.1 (- 0.7 – 0.8)	0.871
Post-intervention	0.6 ± 1.1	0.7 ± 1.4	- 0.1 (- 0.7 – 0.5)	0.724
Change	- 0.5 ± 1.1†	- 0.3 ± 1.4	- 0.2 (- 0.8 – 0.5)	0.602
Shoulder pain (right side) previous 3-months				
Pre-intervention	1.3 ± 2.0	1.3 ± 1.5	0.0 (- 0.8 – 0.9)	0.951
Post-intervention	1.1 ± 1.8	1.6 ± 2.2	- 0.5 (- 1.4 – 0.5)	0.327
Change	- 0.1 ± 2.1	0.4 ± 1.6	- 0.5 (- 1.4 – 0.4)	0.267
Shoulder pain (right side) previous 7-days				
Pre-intervention	0.7 ± 1.7	0.5 ± 1.1	0.2 (- 0.5 – 0.9)	0.529
Post-intervention	0.6 ± 1.6	0.9 ± 1.9	- 0.3 (- 1.1 – 0.6)	0.515
Change	- 0.1 ± 1.9	0.3 ± 1.2	- 0.5 (- 1.3 – 0.3)	0.213
Shoulder pain (left side) previous 3-months				
Pre-intervention	0.8 ± 1.5	0.9 ± 1.5	- 0.0 (- 0.8 – 0.7)	0.913
Post-intervention	0.7 ± 1.3	0.6 ± 1.2	0.0 (- 0.6 – 0.6)	0.969
Change	- 0.2 ± 1.6	- 0.2 ± 1.6	0.1 (- 0.7 – 0.8)	0.895
Shoulder pain (left side) previous 7-days				
Pre-intervention	0.4 ± 1.1	0.5 ± 1.3	- 0.1 (- 0.7 – 0.5)	0.694
Post-intervention	0.4 ± 1.0	0.0 ± 0.2	0.3 (0.0 – 0.7)	0.049*
Change	- 0.1 ± 1.3	- 0.5 ± 1.3†	0.5 (-0.2 – 1.1)	0.149

Values are presented as mean ± SD. Significant between-group-difference is denoted by *. Significant within-group-change is denoted by †. Exercise-training-group (ETG), Reference-group (REF)

#### Pain intensity and sensitivity

Self-reported pre-intervention intensity of neck pain previous 3-months for the whole group was: 2.2 ± 1.8. Pre-intervention pain sensitivity as assessed by PPT in TRA and UNE (mean of right and left) was: 429 ± 182 kPa and 358 ± 160 kPa in ETG, and 411 ± 166 kPa and 334 ± 151 kPa in REF. No significant between-group-differences were found regarding pain intensity or PPT measurements pre-intervention (Tables 2 and 3).

**Table 3:** Intention-to-treat analysis for pressure-pain-threshold in neck and shoulder muscles

	ETG (*n* = 35)	REF (*n* = 34)	(95% Conf. Interval)	*p*-value
Trapezius m. (right)				
Pre-intervention (kPa)	424 ± 187	416 ± 177	8 (-79 – 96)	0.851
Post-intervention (kPa)	409 ± 183	342 ± 143	67 (-12 – 146)	0.094
Change (kPa)	-15 ± 126	- 74 ± 137†	59 (-4 – 122)	0.067
Trapezius m. (left)				
Pre-intervention (kPa)	434 ± 188	405 ± 163	28 (-56 – 113)	0.507
Post-intervention (kPa)	381 ± 169	332 ± 154	49 (-29 – 127)	0.211
Change (kPa)	-53 ± 113†	-74 ± 119†	21 (-35 – 77)	0.456
Upper neck extensors (right)				
Pre-intervention (kPa)	345 ± 157	334 ± 147	11 (-62 – 84)	0.759
Post-intervention (kPa)	347 ± 152	295 ± 116	53 (-12 – 118)	0.110
Change (kPa)	2 ± 118	-39 ± 97†	41 (-11 – 93)	0.117
Upper neck extensors (left)				
Pre-intervention (kPa)	371 ± 170	335 ± 163	36 (-44– 116)	0.377
Post-intervention (kPa)	348 ± 146	292 ± 121	56 (-8 – 121)	0.086
Change (kPa)	-23 ± 110	-44 ± 104†	21 (-31 – 72)	0.424
Reference point				
Pre-intervention (kPa)	643 ± 218	622 ± 231	21 (-87 – 129)	0.700
Post-intervention (kPa)	587 ± 209	553 ± 224	34 (-70 – 139)	0.513
Change (kPa)	-56 ± 125†	- 69 ± 209	13 (-69 – 96)	0.746

Values are presented as mean ± SD. Significant within-group-change is denoted by †. Exercise-training-group (ETG), Reference-group (REF)

#### Associated pain factors

Among pilots, the intensity of left shoulder pain previous 3-months was significantly related to age (*p* = 0.040) and flying hours in a fixed wing aircraft (*p* = 0.005). Intensity of neck pain previous 3-months among crew-members was found significantly related to seated height (*p* = 0.007). No significant relation was found between pain intensity, height, weight, BMI, flying hours in a helicopter, or flying hours with NVG for neither pilots nor crew-members.

### Post-intervention

#### Pain intensity

Regarding the primary outcome of intensity of neck pain previous 3-months, no significant between-group-difference or between-group-change, was found post-intervention (Table 2). Only, with regards to the secondary outcome, intensity of left shoulder pain previous 7-days, a between-group-difference was significant. Additionally, a significant within-group-change was found in ETG regarding intensity of neck pain previous 7-days (from: 1.0 ± 1.4, to: 0.6 ± 1.1, change: - 0.5 ± 1.1 (*p* = 0.024)) (Table 2).

#### Pain sensitivity, PPT

Regarding the secondary outcome of PPT no significant between-group-differences or between group change were found post-intervention (Table 3). However, significant within-group-changes were observed for the ETG regarding PPT in the left TRA (change: -53 ± 113 kPa, *p* = 0.009) and for the reference point (change: -56 ± 125 kPa, *p* = 0.012). For participants in REF a significant-within-group change was found for PPT in the TRA right (change: - 74 ± 137 kPa, *p* = 0.003), and left (change: -74 ± 119 kPa, *p* = 0.001), and for the UNE right (change: -39 ± 97 kPa, *p* = 0.024) and left (change: -44 ± 104 kPa, *p* = 0.045). Overall, REF increased pain sensitivity in more sites in the neck and shoulder than the ETG.

#### Training adherence

**Fig. 2:**
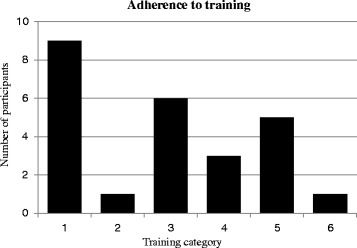
Training adherence; 1) trained regularly 2 - 3 times a week, 2) trained regularly 1 - 2 times a week, 3) trained irregular, but at least 4 times a month (approximately once a week), 4) trained irregularly but at least 2 – 3 times a month, 5) trained some, but stopped training after a while, 6) did not use the training offer

**Fig. 3:**
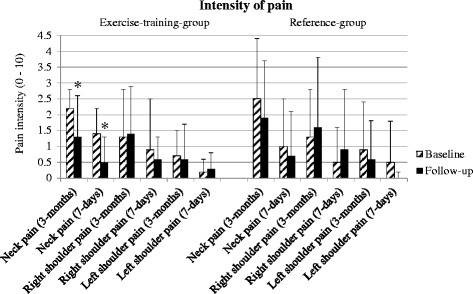
Intensity of neck and shoulder pain analyzed by per-protocol analysis (ETG, n = 10 and REF, *n* = 34). Values are presented as mean ± SD. Significant within-group-changes are denoted by*

Twenty-five participants out of 35 (71.4%) within the ETG returned the post-intervention questionnaire regarding training adherence. Of these, 10 participants (~30%) reported adhering to training regularly between 1-3 times a week throughout the intervention period (Fig. 2). The per-protocol-analysis including participants that adhered regularly to training (*n* = 10) demonstrated no additional significant between-group-differences regarding the primary outcome of intensity of neck pain previous 3-months. Similarly, regarding the secondary outcome values of PPT and additional neck/shoulder pain intensity variables no significant differences were found. However, an additional within-group-change was significant in ETG regarding intensity of neck pain previous 3-months (from: 2.2 ± 0.6, to: 1.3 ± 1.3, change: -0.9 ± 1.0 (*p* = 0.019)) (Fig. 3). Further, intensity of neck pain previous 7-days decreased significantly (from: 1.4 ± 0.8, to: 0.5 ± 0.8, change: - 0.9 ± 1.1 (*p* = 0.029)), in line with results from the intention-to-treat analysis (Table 2).

## Discussion

The main finding of this randomized controlled trial was that 20-weeks of strength, endurance, and coordination training, targeting the neck and shoulder muscles, provided no significant effect on intensity of neck pain previous 3-months, i.e. our primary hypothesis was rejected. However, of major interest was the finding of a high prevalence of neck pain among helicopter pilots and crew-members. Furthermore, a significant secondary outcome regarding a within-group-effect of reduced pain intensity and sensitivity, particularly in ETG, was found.

### Pain intensity

The primary outcome of intensity of neck pain previous 3-months was lower than expected compared with previous studies. Ang et al. previously reported the 3-months intensity of neck pain among helicopter pilots equal to 4.4 using the Borg Category-Ratio scale (CR 0-10 scale) [2]. The scale used by Ang et al. is equal to the one used in our study and results are therefore comparable. Intensity of pain was higher in the study by Ang et al. as compared to our results. Participants within our study constituted the majority of helicopter pilots and crew-members within the RDAF and are therefore considered representative in relation to other helicopter pilots and crew-members. In the study by Ang et al. only pilots who reported pain as “once or a few times over the previous 3-months” were included in the analysis [2]. In comparison, no pain threshold was used to identify pilots or crew-members in our study. This was a deliberate decision to assess if the exercise intervention would be useful in relation to pain prevention. Undoubtedly, this decision has influence on the average intensity of pain among participants. Notably, a decrease in pain is less likely when a low baseline pain level is present due to the “diluting” effect of participants with no pain.

### Pain sensitivity, PPT

Regarding the secondary outcome of PPT the exercise intervention provided no significant between-group-differences. However, a significant reduction in PPT was observed in REF in both TRA and UNE from baseline to follow-up. In comparison, PPT was only significantly reduced in left TRA in ETG. It should be emphasized, that a reduction in PPT is equal to an increase in pain sensitivity. Based on our results, the exercise intervention may therefore have had some impact on pain sensitivity in the neck and shoulder muscles. The exercise intervention was planned during the winter period that includes many flight hours with NVG due to low light conditions. Based on previous studies, demonstrating the impact of head-worn mass by NVG, the winter months must be regarded as the most stressful to pilots and crew-members with respect to cervical load. The exercise intervention may therefore have provided some form of pain relief as pain sensitivity in the neck and shoulder muscles among participants in ETG did not undergo the same significant increase as observed in REF. This needs further investigation as no significant between-group-differences were present. To our knowledge, no previous studies have measured PPT in neck and shoulder muscles among helicopter pilots and crew. However, exercise has been shown to increase pressure-pain-threshold in neck muscles in response to short [26, 27] and longer training interventions [28]. Pressure pain threshold may be considered a “semi-objective” measurement of pain sensitivity because participants mark, in a blinded setting, the incidence of pain occurrence, while increasing pressure is being applied, resulting in the reading of the actual numerical outcome of pressure when the threshold is met [29]. The participants may have underreported the sensation of pain because pain complaints may be regarded as a sign of weakness among military pilots [24]. This would certainly impact the study’s potential regarding additional increases in PPT.

### Pain prevalence and duration

Neck pain was highly prevalent among participants with a 1-year prevalence amounting to 90.3% for pilots and 81.6% for crew-members, respectively. Our results correspond with those of other studies surveying the 1-year prevalence of neck pain among helicopter pilots and crew-members. Van den Oord et al. previously reported a 1-year prevalence equal to 43% for Dutch military helicopter pilots [30] and 62% for crew-members [5]. In comparison, Birger et al. reported a 1-year prevalence of 48% among British helicopter pilots [31]. Our prevalence rates are higher compared to these previous studies. However, discrepancies may be explained by use of different pain definitions and pain cut points. Van den Oord et al. defined pain as “any pain, ache or discomfort” using four pain categories (never, occasional, regular, or continuous) [5, 30]. In the study by Birger et al, no distinctions were made between occasional, regular, or continuous neck pain [31]. In comparison, we also defined pain as “any pain” within our study but used six pain categories (0 days, 1-7 days, 8-30 days, 31-90 days, >90 days, every day). The use of additional pain categories may attribute to the higher 1-year prevalence for any pain among pilots and crew within our study. Based on fairly homogeneous neck pain definitions, the 1-year prevalence within the general adult population (17 - 70 years of age) has been reported to range between 17% - 75% with a mean of 37% [3]. The prevalence of neck pain among helicopter pilots and crew-members must therefore be regarded as high with potential impact on individual health and overall well-being [3] and interference with flying [2]. Neck-pain among helicopter pilots and crew has previously been described as chronic [1]. Our data, however, do not support this definition as the majority of pilots (87.1%) and crew-members (73.7%) in our study reported pain durations of between one and thirty days within the last year. In comparison, only 3.2% of pilots and 2.6% of crew-members reported more than 90 pain days. Adhering to the definition of “chronic pain”, the sensation of pain must be persistent with a duration of three months or more a year [32]. Based on our findings, neck pain among this occupational group may be more appropriately described as “episodic neck pain”. Interestingly, pilots and crew-members have been found reluctant to report pain due to fear of flying restrictions [33] and jeopardize future employment opportunities or pension entitlements [2]. Lastly, pilots and crew fulfilling the highest demands of health in the RDAF will report a lower intensity of pain, as opposed to studies conducted on patient groups seeking medical treatment for pain symptoms [34]. Future studies may obtain valuable knowledge by considering measuring the influence of neck pain, in comparison to pain intensity itself, within this specific occupational group.

### Training adherence

Adherence to training is a challenge and affects the results of an intervention. The lack of a clear intervention effect on pain intensity and PPT may therefore have been influenced by adherence, as only ~ 1/3 of participants in ETG trained regularly. Previously, self-reported adherence to training and actual training participation has been found reliable [35, 36]. However, adherence was analyzed according to 71.4% of questionnaire respondents, leaving some uncertainty regarding transferability to the remaining 28.6% in ETG. In a previously conducted randomized controlled trial aiming to reduce neck pain among Swedish helicopter pilots, 77% of participants adhered to the prescribed daily regime during a 6-week intervention period [37]. In addition, in a former 12-week exercise study on helicopter aircrew within the Canadian forces, aiming to improve neck muscle function, regular training adherence was achieved in 52.8% of participants randomized to neck endurance training, and in 76.1% of participants randomized to neck coordination training [38]. Finally, a recently conducted exercise intervention, aiming to reduce neck and shoulder pain among Danish fighter pilots, 58% of participants adhered to training 1-3 times a week throughout a 24-week intervention period [24]. When compared to our results (28.6%), adherence within our study must be regarded as low. Exercise training must be performed regularly to prove beneficial and the low level of adherence within our study may not be sufficient to conclude on the full effectiveness of the exercise intervention itself. Previously, the importance of supervised training to enhance compliance among aircrew has been underlined [39]. However, uncertainty still exists regarding the benefit of supervised training in general. As demonstrated by Gram et al., 20-weeks of physical exercise training at the workplace (one hour per week) may be highly effective in reducing neck pain, independent of the level of supervision [40]. Due to logistical reasons, supervision was not optional within our study. Pilots and crew have very dynamic work schedules and work on different air bases placed throughout the country, and we therefore designed the intervention to be self-administered. Future studies may have to put effort on how pilots and crew may be followed up frequently, if adherence to training is to be increased, e.g. by the use of information and communication technologies.

### Limitations

Some limitations of the present study must be considered when interpreting data and results. The low level of regular training adherence within ETG must be regarded as a primary limitation. Low adherence reduces the statistical power of the study, and may therefore also have undermined the intervention and increased the risk of erroneous conclusions and consequently a false rejection of an effective exercise intervention. Also, pilots and crew members who had left their flying duties or the military, perhaps because of severe neck pain problems, are not included in this study. The study included subjects with and without neck pain to investigate if specific neck training could reduce the incidence of flight related neck pain. However, including subjects without pain at baseline will impact on the possible overall level of pain within groups, and also the potential to reduce pain. It may be argued that a measurement of flight-related pain would have proven better than a questionnaire assessing intensity of pain previous 3-months and 7-days. Flight related neck pain has been found to develop during and after sorties, and typically last for a day or two. Therefore, it would have been interesting to measure intensity of pain the day after each sortie within the intervention period in order to provide a better understanding of the pain patterns in relation to type of sortie. However, this was not performed in the present study.

## Conclusions

The exercise intervention demonstrated some preventive properties as some neck pain intensity and sensitivity improved within ETG but not within REF. However, no significant between-group-differences were present. The lack of a clear intervention effect on pain intensity may be due to low adherence as only ~ 1/3 of subjects in the ETG engaged in regular training, which may be due to the self-administration of the training regime.

## Abbreviations

BMI: Body mass index
ETG: Exercise-training-group
NVG: Night vision goggles
PPT: Pressure-pain-threshold
RDAF: Royal Danish Air Force
REF: Reference-group
SD: Standard deviation
TIA: Anterior tibialis muscle
TRA: Trapezius muscle
UNE: Upper neck muscles
